# Transcriptomic Profiling of Dental Tissue-Derived Mesenchymal Stem Cells

**DOI:** 10.1155/sci/4789882

**Published:** 2025-10-02

**Authors:** Sema S. Hakki, S. Buket Bozkurt, Zehragul Ergul, Erdal Karaoz, Seyit Ali Kayis

**Affiliations:** ^1^Department of Periodontology, Selcuk University, Konya, Türkiye; ^2^Department of Biochemistry, Nigde Omer Halisdemir University, Nigde, Türkiye; ^3^Center for Stem Cell and Regenerative Medicine, Liv Hospital, Istanbul, Türkiye; ^4^Department of Histology, Istinye University, İstanbul, Türkiye; ^5^Department of Biostatistics, Abant İzzet Baysal University, Bolu, Türkiye

**Keywords:** dental tissues, MSCs, PENK mRNA, periodontal ligament, pulp, transcriptomic analysis

## Abstract

The aim of this study was to compare whole-genome gene expressions of periodontal ligament (PDL) and pulp (P) mesenchymal stem cells (MSCs) isolated from third molar (m), premolar (p), and deciduous (dec) teeth. Total RNAs were isolated and used for cRNA synthesis. Human Expression Hybridization Assay was used for 47,000 probes. Data were subjected to quantile normalization before analysis. Based on the differentially expressed genes, immunomodulation properties of m/p/dec-MSCs were evaluated. Lymphocytes cocultured P/pdl-MSCs were investigated for apoptosis and cell survival of phytohemagglutinin-stimulated T cells. T cells and medium supernatants were collected on Days 1 and 4 of the experiments to evaluate T-cell proliferation by WST-1 and apoptotic markers by flow cytometry. Statistical analysis demonstrated that 291 genes were differentially expressed ≥2 fold in the cells isolated from p/m/dec, and pdl/P MSCs. The most significant difference was recognized in the proenkephalin (PENK) gene (24-fold) in pPDLMSCs, epidermal growth factor-like protein 6 (EGFL6), and complement factor D (CFD) genes were differentially expressed in decPMSCs 16.9-fold and 11-fold, respectively, when compared to other MSCs. A difference in PENK mRNA expression was also confirmed by RT-PCR. Findings of the study revealed that all dental MSCs cocultured with T cells suppressed the proliferation of T cells on Day 1 when compared to T cells alone (*p*=0.001). The suppression of T lymphocytes proliferation, PENK, and IL-10 mRNA expressions was higher in pPDLMSCs. Highest PENK and IL10 mRNA expressions and T-cell regulation in PDLMSCs suggested that PDLMSCs might be a promising candidate for immune regulation.

## 1. Introduction

Mesenchymal stem cells (MSCs) are widely used in regenerative dentistry due to their dual roles in tissue regeneration and immunomodulation [1–3]. Numerous in vivo studies have demonstrated their potential for both hard and soft tissue regeneration [4–8]. However, identifying the most suitable MSC source for specific clinical applications remains challenging. Beyond their regenerative roles, MSCs play a pivotal part in inflammation resolution, which is crucial in the wound healing cascades [9, 10]. MSCs derived from dental tissues, including pulp (P), periodontal ligament (PDL), gingiva, alveolar mucosa, and deciduous (dec) teeth, have shown promise in this context. Notably, differences in differentiation potential and gene expression profiles have been observed between MSCs from P and PDL [11, 12].

Functional differences in MSCs isolated from human dental P (hDP) and human PDL (hPDL) were found regarding their differentiation potentials [13]. Differences were not only in the differentiation potentials, but also in transcriptional level differences were demonstrated using MSCs-specific arrays [11]. Previous studies have reported that PDL–MSCs express higher levels of immunoregulatory molecules such as HLA-G and IL-10 compared to P-derived MSCs [14, 15] while others demonstrated that P, gingiva, and pdl-MSCs showed similar immunoregulatory properties to those of bone marrow, regarding the inhibition of cellular proliferation of CD4+- and CD8+-activated T cells [16].

Proenkephalin (PENK), a gene implicated in IL-10 upregulation, anti-inflammatory signaling, and neuropeptide synthesis, may play a critical role in MSC-mediated immune regulation [17]. It was demonstrated that the intrathecal administration of PENK gene-transduced bone marrow MSCs (BMSCs) can effectively relieve pain and might be beneficial in pain of humans [18, 19]. It was also found that PENK expression is selectively increased in mineralized cultures and formation of bone required PENK expression [20, 21].

The present study aims to (1) compare transcriptomic profiles of MSCs derived from third molar (m), premolar (p), and dec teeth (from both P and PDL), (2) validate key genes such as PENK and IL-10 using real-time PCR, and (3) evaluate the immunomodulatory effects of these MSCs on T-cell proliferation and apoptosis in coculture settings.

## 2. Materials and Methods

### 2.1. MSCs Isolation

The m and p teeth were extracted for orthodontic reasons, and the dec teeth were extracted due to permanent teeth eruption. Ethical approval of the study was taken from Selcuk University. While PDL and P tissues were isolated from p (n:3) and m teeth (n:3), only P tissue was isolated from dec teeth (n:3). MSCs characteristics were checked by flow cytometry for cell surface markers and the differentiation to three different lineages [1].

### 2.2. RNA Extraction

Total RNA was extracted from each cell using an RNA isolation kit1 compatible with the Human HT-12 v4 Expression Bead Chip Direct Hybridization Assay kit2 following manufacturer instructions at 6 h. After that, the quality and quantity of RNA were determined at absorbance 260 and 280 nm by NanoDropTM 1000. 3 RNA with 260/280 ratios in the range between 2.0 and 2.3 were used in this study. To confirm the quality of RNA samples, concentration and integrity of RNA were accessed by BioAnalyzer 2100 Agilent with the Agilent RNA 6000 nano Kit. 2 Quality was evaluated using the RNA integrity number (RIN) value, that determined for intact 18S and 28S ribosomal peaks. The RNA samples with 18S and 28S ribosomal peaks and RIN between 7 and 10 were used in the microarray experiments.

### 2.3. Microarray Analysis

Using the high-throughput Illumina Human HT-12 v4 Expression Bead Chip Direct Hybridization Assay,^2^ the whole transcript expression levels of about 47,000 target genes were determined. Total RNA from cells isolated m/p/dec teeth and P/pdl were used for microarray analysis assays. To produce cDNA, high-grade total RNA was needed. Amplification and labeling of complementary RNA with biotin was performed using the TargetAmp-Nano Labeling Kit for Illumina Expression BeadChip2 Labeled cRNA was hybridized to HumanHT-12, V4 BeadChip arrays,^4^ followed by staining with streptavidin-Cy35 to visualize. The arrays were washed using Illumina high-stringency wash buffer for 30 min at 55°C, followed by scanning according to standard Illumina protocols. The arrays were scanned using iScan systems. The Bead Array Reader was analyzed using GenomeStudio Software. 4

### 2.4. Evaluation of Selected Genes Expressions Using Real-Time PCR

Total RNA from cells isolated m/p/dec teeth and P/pdl were isolated using a monophasic solution of phenol and guanidine isothiocyanate6 after 6 h. Then total RNA was reversed transcribed into cDNA using RevertAid First Strand cDNA Synthesis Kit,^7^ following the manufacturer's protocol. To validate mRNA expression and explore the potential immunomodulatory potential of cells, the PENK (24-fold) gene, that the most significant difference among 291 genes differentially expressed in the cells isolated from p/m, and pdl/P MSCs was further analyzed by real-time PCR assay. The specific transcripts of PENK and IL-10 were measured by RT-PCR using the universal probe library (UPL) probes^8^ and analyzed with Stratagene MX300P. 9 The assay IDs of the UPL probes used are as follows: PENK (UPL Assay ID: 111450); IL-10 (UPL Assay ID:). Glyceraldehyde 3-phosphate dehydrogenase (GAPDH) was used as the housekeeping gene control. For the RT-PCR analysis, 1.0 μL of cDNAs was used per 25 μL of final reaction volume in a thermal cycler. The amplification profile for PENK, IL-10, and GAPDH was 95/600; 95/15; 57/60 (temperature in °C/time in seconds), and 35–40 cycles.

### 2.5. Immunomodulation Experiments

#### 2.5.1. T Cells Isolation

Peripheral blood (PB) samples were obtained from a healthy female volunteer (donor age is 24). T cells were isolated from blood and cultivated in T-25 flasks in a 5% CO_2_ atmosphere at 37 °C in a culture medium composed of RPMI-1640 medium,^10^ 15% FBS, 100 U/mL penicillin, 0.1 mg/mL streptomycin, 200 mM glutamax,^10^ and 2% mitogen phytohemagglutinin10 for 48 h.

#### 2.5.2. Coculturing MSCs With T Cells

To generate an adherent cell layer, hDP and hPDL MSCs were seeded 24 h earlier in a six-well plate (3 × 10^5^ cells/well). The two media used were optimized for each cell type: RPMI-1640 (with PHA) for T lymphocyte activation, and DMEM/F12 for maintaining MSC viability. Coculture was performed using a hybrid medium containing a 1:1 mixture to support both cell types without compromising functionality. The experiments entailed PHA-stimulated T cells (PHA-T cells) only (T-cell control) and hDP and hPDL MSCs/PHA-T cells (1:1). The culture wells were separated with transwell inserts^11^ with a 0.4 µm pore size to allow exchange of soluble factors while preventing cell migration; PHA-T cells were cultured in upper chambers and MSCs in lower chambers for 1 and 4 days. T cells and medium supernatants were collected on Days 1 and 4 of the experiments to evaluate T-cell proliferation by WST-1 and apoptotic markers (Annexin V-PI) by flow cytometry.

#### 2.5.3. Proliferation Detection by WST-1 Assay

To evaluate immunomodulatory effects of MSCs on T cells, after coculture, collected PHA-T cells (1 × 10^5^ cells/well) were plated in triplicate on 96-well plates with 10 µL 2-(4-Iodophenyl)-3-(4-nitrophenyl)-5-(2,4-disulfophenyl)-2H-tetrazolium (WST)-1^12^ added into each well. The plate was incubated at 37°C in a humid atmosphere containing 5% CO_2_ for 3 h. The optical density (OD) value of samples was measured with a microplate enzyme-linked immunosorbent assay (ELISA) reader13 at a wavelength of 480 nm.

#### 2.5.4. Apoptosis Detection by Flow Cytometry

After coculture, collected PHA-T cells treated with Annexin and PI antibodies. After incubation, the flow-cytometry analysis was performed using FACS-Calibur,^14^ and the results were evaluated using the BD CellQuestTM software program.

### 2.6. Statistical Analysis

The WST-1 experiments were performed twice to ensure reproducibility. Before statistical analysis, distributional properties of variables were evaluated using the Shapiro–Wilk test. Normally distributed variables are analyzed using one-way ANOVA, and a post hoc test is performed via the Tukey HSD test. Log transformation was applied to stabilize the variance of the apoptosis, and analysis was performed on the transformed data. Transformation was not adequate for Wst Day 4. Consequently, the Kruskal–Wallis test was applied to compare groups for it.

The gene expression data normalization process was performed via the 2^−Δ*C*_*T*_′^ method where Δ*C*_*T*_′=*C*_*T*, target_ − *C*_*T*, reference_, here, *C*_*T*, target_ and *C*_*T*, reference_ are the threshold cycles for the target and reference gene amplification, respectively. The averages of the technical replicates of the normalized data of the groups were compared with a Mann–Whitney *U* test.

## 3. Results

### 3.1. Identification of Expressed Genes Related to Immunomodulation of m/p/dec-MSCs

**Figure 1:**
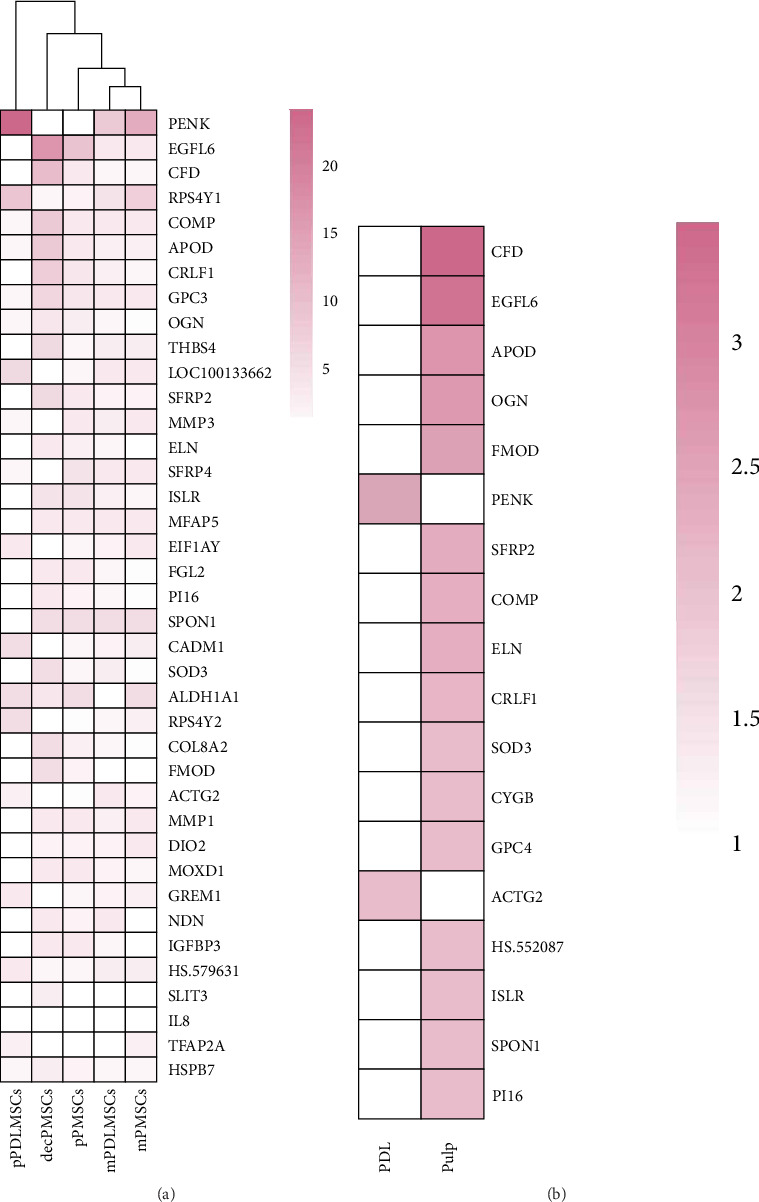
(A) Differential gene expressions of periodontal ligament (PDL) and pulp (P) mesenchymal stem cells (MSCs) isolated from third molar (m), premolar (p), and deciduous (dec) teeth. For each gene white color group is the reference group. The intensity of color in other groups increases with the increasing differentially gene expression level, and the color scale shows the fold change (FC). Genes with over 4 FC are included. Hierarchical cluster heat maps were produced by using Euclidean distance. (B) Differential gene expressions of periodontal ligament (PDL) and pulp (P) mesenchymal stem cells (MSCs). For each gene white color group is the reference group. The intensity of color in the other group increases with the increasing differentially gene expression level, and the color scale shows the FC. Genes with over 2 FC are included.

Gene expression levels in p/m/dec and pdl/P MSCs were analyzed in comparison with among themselves. In total, 291 genes were differently expressed ≥2.0 (*p*-value  < 0.05) a fold change in p/m/dec and pdl/P MSCs. From the unsupervised hierarchical clustering analysis with 24-fold change (*p*-value  < 0.05), 16.9 fold change (*p*-value  < 0.05), 11 fold change (*p*-value  < 0.05), the heat map represented three genes upregulated and 288 genes downregulated in p/m/dec and pdl/P MSCs when compared with the among themselves (Figure 1A,B). Among these, PENK was the highest upregulated at 24-fold change in pPDLMSCs, while epidermal growth factor-like protein 6 (EGFL6) and complement factor D (CFD) genes were the highest upregulated at 16.9 and 11-fold change in decPMSCs. The up- and downregulated genes in p/m/dec and pdl/P MSCs are shown in Table 1.

### 3.2. Quantification of Selected Genes Using Real-Time PCR

**Figure 2:**
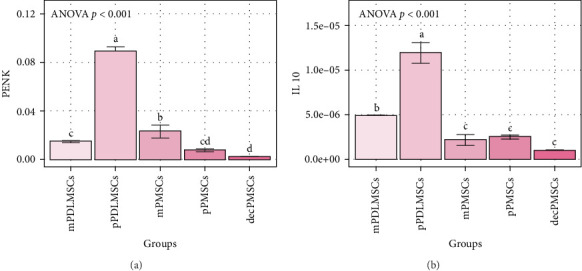
Bar graphs of (A) PENK and (B) IL10. In both graphs, bars show differentially gene expression means for the groups, and error bars show their standard deviations. *p* Values were obtained via one-way ANOVA. For each graph, bars that do not share the same letters have statistically significantly different means determined by the Tukey test.

Real-time reverse transcriptase polymerase chain reaction was performed using UPL probes to validate the finding of the PENK gene in microarray analysis and IL-10. Higher PENK expression was observed in pPDLMSCs compared with m/dec, and pdl/P MSCs (*p* < 0.01) (Figure 2A,B). Similarly, pPDLMSCs showed significantly higher IL-10 expression compared with m/dec, and pdl/P MSCs (*p* < 0.01).

### 3.3. The Results of Immunomodulation Experiments

**Figure 3:**
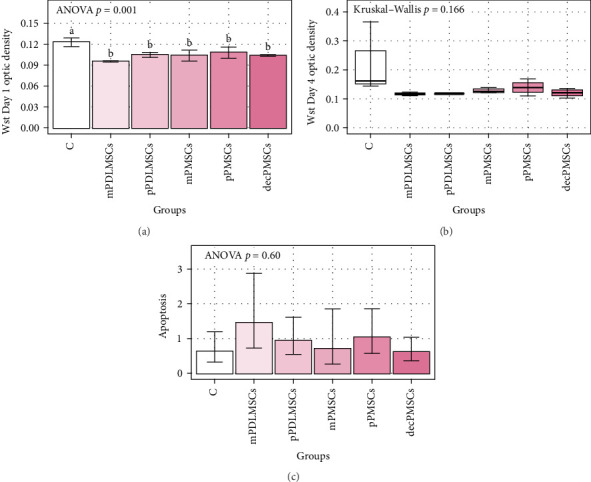
(A) Bar graph of Wst Day 1 optic density. Bars show means for the groups, and error bar shows their standard deviations. ANOVA *p* was obtained via oneway ANOVA. Bars that do not share the same letters have statistically significantly different means determined by the Tukey test. (B) Boxplots of Wst Day 4 optic density for groups. (C) Bar graphs of apoptosis. ANOVA *p* was obtained via one-way ANOVA by performing on the log-transformed values. Thus, bars show geometric means, and error bars are geometric standard deviations.

The findings of the experiments, including coculturing MSCs with T cells, showed that p/m/dec and pdl/P MSCs cocultured with T cells suppressed proliferation on Day 1. MSCs induced apoptosis of T cells when compared to T cells alone, but this was not statistically significant (*p* > 0.05). However, PDL-derived MSCs seem to supressed T-cell proliferation more than P-derived MSCs on Day 4, but the difference was not significant (Figure 3A–C).

## 4. Discussion

MSCs are major actors in the reprograming and resolution of the inflammation. Wound healing starts always with inflammation. Hence, the period of the resolution of the inflammation is critical to determine the healing type. If the resolution of the inflammation period lasts longer, the regeneration or repair capacity of the tissues is affected negatively. MSCs are not only the active players in the regeneration process but also regulate inflammation [22]. Periodontal tissues are highly exposed to the environmental factors, including microbial dental plaque, trauma, occlusal parafunctions, smoking, etc. These factors are complicative during wound healing not like in other tissue's healing. Hence, the reprograming of the inflammation during periodontal healing is critical to achieve periodontal regeneration instead of repair.

In the literature, according to the best knowledge, there is no study comparing MSCs isolated from different teeth, like m vs. p. In our previous study, we compared PDLMSCs vs. PMSCs isolated from p teeth using an MSCs-specific array, and we reported differences in genes regarding their regenerative potential and immune-regulation functions. Results of our previous study demonstrated that pdl-MSCs expressed higher HLA-G and IL-10 mRNA expressions, functioning for immune-modulation compared to PMSCs. We concluded that pdl-MSCs are promising candidates for immune-modulation function instead of PMSCs. Thus, we decided to compare MSCs not only P vs. pdl but also m vs. p vs. dec teeth using whole genome analysis. Our results confirm the immunomodulatory potential of MSCs derived from dental tissues, particularly PDL-MSCs. The suppression of T-cell proliferation observed on Day 1 of coculture, and higher PENK and IL-10 mRNA expression levels in PDL-MSCs, support the hypothesis that these cells may possess superior immune-regulatory properties compared to P-derived MSCs.

In the present study, a 25-fold change in the PENK gene was observed in PDLMSCs isolated from p teeth. Hence, we focused more on the PENK gene which we are not aware of enough its function in MSCs. It was reported that PENK suppresses Th1 cytokine expression and macrophage activation by stimulating secretion of IL-10 anti-inflammatory cytokine. After transcriptomic analysis, we evaluated PENK and IL-10 mRNA expression by quantitative PCR, and whole-genome analysis results were confirmed. After confirmation of the PENK and IL-10 mRNA expression, we exposed MSCs isolated from different regions of different teeth with stimulated T lymphocytes and evaluated their proliferation and apoptosis. Decreased T-cell proliferation was observed in all types of MSCs when compared to stimulated T lymphocytes. Moreover, the decrease in cell proliferation was higher in PDLMSCs in comparison to PMSCs, but not statistically significant. There was no significant difference for annexin, which represents apoptosis of the cells. It was concluded that MSCs isolated from dental tissues have the potential to regulate T-cell proliferation. While the coculture results were promising, the observed differences in T-cell apoptosis and proliferation were not statistically significant on Day 4. This may reflect limitations in sample size or donor variability, which are known factors in MSC-T-cell interaction assays. Further studies with larger sample groups or animal models may provide more robust conclusions.

Higher PENK and IL-10 gene expressions suggested that PDLMSCs might be a good candidate for immunoregulatory properties when compared to PMSCs. Dental tissue-originated MSCs are promising to treat autoimmune diseases by suppressing the immune system. Although PMSCs were more attractive cells in the treatment of autoimmune diseases in the literature [23], PDLMSCs could be also considered a promising MSC source for immune-regulation studies.

In the study of Yang et al. [24], heterotopic ossification-related genes were investigated using BMSCs from heterotopic ossification tissues. They identified 10 hub genes for heterotopic ossification, including CX3CL1, CXCL1, ADAMTS3, ADAMTS16, ADAMTSL2, ADAMTSL3, ADAMTSL5, PENK, GPR18, and CALB2. PENK was one of the hub genes in heterotopic ossification [24]. In the present study, PENK was one of the most differentially expressed genes in the whole genome array when we compared P and PDL-derived MSCs. We may assume that PDL-derived MSCs have the potential to induce bone formation in comparison to P-derived MSCs, and that PENK gene expression might be critical in the MSCs of PDL in maintaining the periodontal tissues.

Min et al. [23] reported 11 clinical trials demonstrating the feasibility and safety of PMSCs in various diseases. Allogeneic PMSCs seeded scaffold application reduced periodontal pockets and increased the bone formation in periodontal intrabony defects without any side effects [25]. Safety and efficacy of PMSCs were also reported in the treatment of patients with severe COVID-19 [26]. The long-term efficacy and safety of MSCs in type I diabetes mellitus, systemic lupus erythematosus, osteoarthritis, and ulcerative colitis have been still investigated. However, PMSCs have been more extensively investigated when compared to PDLMSCs in immune-mediated diseases. Hence, comparison of the cells isolated m/p/dec teeth and PDL vs. P should be explored more to have optimized findings in stem cell-based therapies.

Further research is warranted to confirm other differentially expressed genes at the mRNA and protein levels. Immunoregulatory functions of MSCs should be tested in animal experiments for potential usage in medicine. Additional studies are also required to clarify other immune-suppression-related genes and pathways of dental MSCs. Although we used microarray-based transcriptomics, which remains a reliable and validated approach, we acknowledge that next-generation sequencing (RNA-seq) would provide greater resolution and help identify novel immunomodulatory transcripts. Future work using RNA-seq is warranted to expand upon our current findings. In this study, we did not validate the overexpression of PENK and/or IL-10, and did not perform functional knockdown or overexpression studies. Such experiments (e.g., siRNA-mediated silencing of PENK) would be essential to causally link gene expression with immunomodulatory effects.

One of the most differentially expressed genes in our dataset was PENK, which showed higher expression in MSCs derived from PDL compared to other dental tissues. PENK, a precursor of enkephalins involved in neuroimmune signaling and immune cell regulation [27, 28]. It has also been reported that PENK secreted by placental MSCs can inhibit fibroblast activation, suggesting an active paracrine role in tissue modulation [29]. These findings are consistent with previous studies indicating that dental MSCs exert immunomodulatory effects through secreted factors [30, 31].

Supporting this observation, our Gene Ontology (GO) and Kyoto Encyclopedia of Genes and Genomes (KEGG) pathway enrichment analysis revealed significant terms related to extracellular matrix organization, secretory granules, and immune response processes [32]. These pathways align with PENK's biological profile as a secreted neuropeptide capable of modulating the local immune environment. PENK-related functional pathways are shown in Figure S1. While our study does not yet provide direct functional validation of PENK's role, its strong expression in immunomodulatory MSC populations and its association with relevant biological pathways make it a promising candidate for further investigation.

## 5. Conclusions

The immunoregulation properties of MSCs capability are affected by the donor's age and gender (24). Other factors effecting these capability are source type and region of the organ. Hence, to optimize the effect of MSCs application in clinical treatment, associated factors or determinant genes for immunoregulatory potentials of MSCs cells should be further investigated. PENK and IL10 might be considered determinant genes for immunoregulatory potentials of stem cells. In conclusion, PENK and IL-10 may serve as signature genes for identifying immunoregulatory MSCs from dental tissues. While PMSCs have received more attention in clinical trials, our results suggest that PDLMSCs deserve further investigation and may be strong candidates for applications in immune-mediated disorders.

## Figures and Tables

**Table 1:** List of the genes with over two-fold differential expression in one of the MSCs sources.

Rank	Gene	MolarPDL	PrePDL	MolarPulp	PrePulp	DecPulp
2	PENK	9.026217	24.07002	13.55497	5.110123	1
3	EGFL6	5.427076	1	4.446372	9.979128	16.97639
4	CFD	2.439915	1	2.092451	4.81474	11.01452
5	RPS4Y1	5.198564	9.73364	8.196153	2.925334	1
6	COMP	4.378844	1	3.474918	5.531799	9.283729
7	APOD	3.58275	1	3.368142	6.053441	9.049558
8	CRLF1	3.908217	1	2.647684	4.943412	8.722894
9	GPC3	5.163572	1	3.493061	4.757657	7.313064
10	OGN	2.251888	1	1.879755	4.06348	5.126718
11	THBS4	3.868713	1	3.931871	2.087609	6.737465
12	LOC100133662	3.701338	6.678229	5.259023	2.157939	1
13	SFRP2	3.017272	1	3.189495	4.25524	6.659689
14	MMP3	3.923283	2.173978	4.44073	6.621443	1
15	ELN	2.285921	1	1.355833	3.668252	6.134353
16	SFRP4	6.034635	1	5.438092	4.670627	1.229724
17	ISLR	3.344855	1	2.768896	5.122856	5.610388
18	MFAP5	3.725009	1	2.813448	5.59587	4.379911
19	EIF1AY	3.218216	5.511715	4.30421	2.136658	1
20	FGL2	2.530789	1	1.854764	2.901164	5.150593
22	PI16	2.25781	1	1.583786	3.255426	5.127713
23	SPON1	3.282023	1	3.340318	4.780225	5.096521
24	CADM1	3.206611	5.008532	4.141502	2.0628	1
25	SOD3	1.936395	1	1.752589	2.716224	4.930895
26	ALDH1A1	1	4.326419	5.893268	6.181342	4.919942
27	RPS4Y2	2.474605	4.779333	3.560223	1.804519	1
28	COL8A2	2.430374	1	1.91095	3.405983	4.257817
29	FMOD	1.306646	1	1.292981	2.954624	4.540189
30	ACTG2	4.506339	3.762303	3.247775	1.666156	1
31	MMP1	3.601218	1	4.264209	3.084837	4.503396
32	DIO2	3.227707	1	4.430851	3.031291	3.11453
33	MOXD1	3.147817	1	2.611097	3.883588	4.337843
34	GREM1	2.901718	4.362981	3.476774	2.439464	1
35	NDN	2.119474	1	1.797927	2.925312	4.270999
36	IGFBP3	2.601554	1	1.349303	3.447305	4.240172
37	HS.579631	3.851195	4.237012	2.958123	2.694564	1
38	SLIT3	2.091189	1	1.62464	3.218519	4.192793
39	IL8	2.634505	4.018872	2.401354	1	2.145875
40	TFAP2A	2.537339	3.668118	4.173649	1.830939	1
42	HSPB7	2.592709	1	2.252266	2.913933	4.130544
43	ALPL	2.592951	1	2.111264	3.05725	3.99651
44	HS.552087	1.651884	1	1.483417	2.800194	3.964165
45	C20ORF103	1.680061	3.903185	2.721263	1.366767	1
46	GPC4	1.676039	1	1.851507	2.704593	3.900889
47	GOLPH4	2.654078	3.836819	3.622749	1.810699	1
48	CD1C	2.217088	3.801531	2.712296	1.764632	1
49	GLDN	1.71295	1	2.034681	1.837277	3.755445
50	TUBB2B	1.555702	1	1.164961	1.888785	3.725356
51	PTGIS	2.09953	1	1.700378	2.641752	3.679651
52	PRKAR1A	2.204307	1	1.947398	3.153592	3.667929
53	CLDN11	3.639529	1	1.916562	2.999277	2.972762
54	COL4A1	1.152223	2.709004	1.043369	3.586379	1
55	CYGB	1.540448	1	1.437468	3.060346	3.562357
56	LYPD1	2.559584	3.555769	3.283837	2.130295	1
57	SUSD2	1.037487	2.041806	1	3.542787	1.38278
58	FNDC1	2.22132	1	1.739731	2.818305	3.537831
59	OXTR	3.492529	3.530463	2.670259	2.467888	1
60	PITX1	2.413405	1	1.960947	2.568581	3.523188
62	PCOLCE2	2.103151	1	1.894175	2.612929	3.503399
63	THRA	2.010657	3.426099	2.734886	1.777743	1
64	IGFBP5	2.314658	1	2.646188	2.186654	2.836513
65	PLAC9	1.663706	1	1.563275	2.626587	3.272591
66	SEPP1	1.974432	1	2.368805	2.312189	3.264106
67	HS.25318	2.298111	2.443486	3.25183	1.695597	1
68	DPT	1.477709	1	1.402411	1.845839	3.226997
69	PEAR1	1.896987	1	1.266374	2.527344	3.194422
70	LOC100008588	3.128367	1.692267	2.093004	1	2.336397
71	CPE	1.50279	1	1.617478	2.15472	3.124973
72	CD14	2.542042	1.255247	3.085546	2.46097	1
73	SIT1	1.883472	3.042363	2.252736	1.546641	1
74	AKR1C4	2.03191	1	1.964851	2.34699	3.024665
75	S100A16	1.690546	1	1.30999	1.897239	3.017505
76	COL7A1	1.924092	2.972368	2.58799	1.596375	1
77	CXCR7	1.816734	1	1.842153	2.273911	2.970378
78	PAG1	2.240239	2.782694	2.923643	1.744325	1
79	CAV1	1.903534	2.920361	1.911762	1.805035	1
80	NPTX2	1.556915	2.918864	1.671162	1	1.01392
82	OLFML1	1.843211	1	1.751011	2.575908	2.915511
83	KCNK2	1.534291	2.743253	1.486311	1.752767	1
84	CYP1B1	1.701692	1	1.605287	2.426608	2.88066
85	SFRP1	1.980984	1	1.54402	1.800957	2.875072
86	LYN	1.755459	2.858768	2.162232	1.621969	1
87	CPA4	2.758779	2.851057	1.818188	1.949566	1
88	TNFAIP6	1.126902	1	1.215493	1.863145	2.846945
89	FOXQ1	1.700184	2.194372	2.838673	1.527039	1
90	PAMR1	1.976085	1.861515	2.836767	1.954298	1
91	C1QTNF1	2.242973	2.820402	2.815319	1.610511	1
92	NR5A2	2.058424	1	1.670436	1.767042	2.588256
93	VCAN	1.79908	1	1.584958	2.342529	2.786061
94	RSPO2	1.861762	1	1.6472	2.042925	2.785907
95	ALDH3A1	2.262118	1.39628	2.785009	1.600638	1
96	LRRN3	1.799946	2.302675	2.583625	1.28768	1
97	TMTC1	1.485154	1	1.4245	1.740539	2.741787
98	CYTL1	1.787187	1	1.459384	2.666062	2.740345
99	CD24	2.73701	2.081115	2.320084	1.481413	1
100	TMEM132B	2.06959	2.446596	2.735963	1.566717	1
102	OLFML2A	1.442589	1	1.446924	1.523117	2.729867
103	MAB21L2	2.006141	1	1.717869	2.035888	2.724346
104	LGMN	1.785592	1	1.53994	2.507388	2.53474
105	TMEM158	2.196501	2.134261	2.701883	1.490391	1
106	RBP1	2.673291	1.679712	2.148994	1.186446	1
107	MTSS1	1.721002	2.667647	2.419446	1.099318	1
108	COL14A1	1.098339	1	1.363818	2.031574	2.667526
109	EPB41L3	1.516758	1	1.293772	1.418076	2.663144
110	SGCD	2.170924	1	1.726534	2.665417	3.434202
111	C5ORF23	1.275308	1	1.084705	1.948685	2.657257
112	SERPINA3	1.230156	1	1.166007	2.645865	2.180888
113	CTHRC1	1.820727	2.544429	2.199998	1.754048	1
114	LOXL3	1.577962	1	1.277929	1.9821	2.636744
115	PLOD2	1.697926	2.398528	1.609275	2.024383	1
116	HSPB8	1.524196	1	1.246754	2.006923	2.625167
117	AK5	2.006847	2.623902	2.032638	1.462405	1
118	SVIL	1.772941	1.689111	2.620124	1.469621	1
119	TMEM200A	1.877756	1	1.566783	2.127035	2.609411
120	GAS1	1.598284	2.096695	2.586336	1.156927	1
122	CPXM2	2.58532	1	2.279769	2.11929	1.753078
123	CBLN2	1.673981	1.253776	2.583104	1	1.00147
124	C8ORF13	1.775689	2.580035	1.70594	1.390834	1
125	OMD	1.806375	1	1.831402	2.159101	2.576034
126	HS.444329	1.799048	1	2.02684	1.910284	2.554557
127	XIST	1.699712	1	1.323404	2.029939	2.552668
128	MEIS2	2.05956	1	1.615925	2.049088	2.548876
129	CLDN1	1.69962	2.533233	1.992182	1.466125	1
130	NMB	1.530527	1	1.82044	2.532929	2.394857
131	SLC14A1	1.935395	2.40908	2.519287	1.233335	1
132	SLC7A8	1.91052	1	2.50741	1.714876	1.980783
133	LRIG1	1.421559	2.497129	1.707145	1.158463	1
134	CKB	1.715912	1	1.261061	1.79805	2.48113
135	GLRX	1.616348	2.475928	1.690316	1.757178	1
136	LOC100134073	2.024077	2.438659	2.473265	1.660298	1
137	GAS6	1.89535	2.472337	1.727485	1.465405	1
138	HAS2	1.464416	1.181208	1	1.52857	2.472012
139	NETO2	1.464751	2.470003	1.470528	1.138399	1
140	KBTBD11	1.659693	1	1.666832	1.696712	2.456293
142	MN1	1.899456	1	1.699069	2.140693	2.393035
143	CRIP2	1.769346	1	1.448526	2.093189	2.441594
144	KIAA1199	1.779654	2.439013	1.795459	1.38554	1
145	AMY1A	1.565714	2.436862	1.768655	1.412491	1
146	KYNU	1.423455	1.204417	2.435829	1.052699	1
147	RDH10	1.449398	1	2.069956	1.452224	2.429282
148	CFH	1.240583	1	1.272586	1.778215	2.425952
149	TNC	1.888958	1	1.363379	1.449925	2.479335
150	CYBRD1	1.804209	1	1.859274	1.903931	2.345012
151	LAMA4	1.289954	1	1.330823	1.991854	2.347203
152	TIMP3	1.59664	1	1.537201	1.95569	2.413423
153	C1QTNF5	1.459466	1	1.332842	1.561947	2.411438
154	HMCN1	1.341565	1	1.614949	2.407675	1.886746
155	KAZALD1	1.573045	1	1.509572	1.950989	2.405054
156	MX1	1.754097	1.790134	2.404247	1.588942	1
157	NPTX1	1.237588	2.399711	1.490741	1	1.08905
158	HBEGF	1.210475	1.092466	1	1.30042	2.393654
159	AKR1C2	1.314145	1	1.244965	2.236224	2.391361
160	CYP26B1	1.944548	1	2.380489	1.316354	1.024515
162	KRT34	1.59026	1.369625	1	2.049826	2.375722
163	FGFR3	1.398601	1	1.229232	1.442882	2.374407
164	MEGF6	1.50134	1	1.289218	2.136576	2.372979
165	ITPKA	1.817408	2.161845	2.370132	1.356847	1
166	AKR1C3	1.511339	1	1.634609	2.368671	2.367477
167	MMP10	2.23698	1	2.367911	1.503977	1.171361
168	CCRL1	1.907469	1	1.883617	1.643549	2.365789
169	LUM	1.413409	1	1.448482	2.49507	2.02287
170	HPCAL1	1.66654	2.359618	2.122074	1.484982	1
171	DDX3Y	1.364815	2.359586	1.717632	1.288693	1
172	CCND2	1.433593	1.029545	1.834612	2.357026	1
173	WFDC1	1.515182	1	1.22321	1.654667	2.3505
174	CNTNAP2	1.506025	1	1.99884	1.875451	2.348683
175	FAM167A	1.71751	2.348677	1.558797	1.351644	1
176	FBLN5	1.616379	1	1.210183	1.853075	2.34186
177	FAM43A	1.444956	1	1.29893	2.341455	1.922136
178	PALM	1.649267	1	1.519802	2.231683	2.338911
179	LOC100008589	1.754397	1.313273	1	1.146581	2.324874
180	TGFB3	1.245706	1	1.732782	2.030077	2.322149
182	MGP	1.30411	1	1.352482	1.776372	2.320542
183	LOC644396	1.097556	1	1.043466	1.641474	2.312315
184	HAPLN1	1.653484	1	1.138509	1.678278	2.309826
185	BEX1	1.004452	2.278833	1.002358	2.307578	1
186	PTGS1	2.271039	1	1.766641	2.288846	3.07669
187	UHRF1	1.715269	2.296966	1.401798	1	1.328581
188	NFE2L3	1.656382	2.294989	2.069657	1.205355	1
189	FBXO32	1.696753	1.323536	2.291616	1.272455	1
190	LOC651872	1.894675	1	1.858098	1.576224	2.281819
191	PPP1R3C	1.819588	1	1.532403	2.280854	2.19157
192	SPON2	1.558129	1.807776	2.278777	1.847801	1
193	INA	1.623977	2.267005	2.02404	1.365403	1
194	MGST2	1.656331	1	1.537949	2.015527	2.262973
195	ITGA3	1.535503	2.255768	1.605122	1.6527	1
196	SPRY1	1.694683	1	1.994352	1.742077	2.245296
197	GDF10	1.300751	1	1.203359	2.244828	2.102539
198	FAM107A	1.537391	1	1.354268	1.449709	2.24262
199	LOC645676	1.537858	2.23693	1.74466	1.363992	1
200	LOC646628	1.298916	2.236466	1.580745	1.181529	1
202	HS.34447	1.585365	2.231371	1.712054	1.281497	1
203	CDH13	1.082081	2.223876	1.171576	1.763111	1
204	LOC728255	1.70204	2.216467	1.434624	1.470436	1
205	LIMCH1	2.061564	1	1.385907	2.097768	2.210144
206	WDR41	1.469083	1	1.210725	2.137488	2.206553
207	LOC646821	1.295157	1.702474	1	1.562454	2.204059
208	NOTCH3	1	1.355139	1.081623	2.203261	1.711497
209	ZNF521	1.698136	1	2.20261	1.764238	2.033284
210	PCDH18	1.793373	1	2.199232	1.471736	1.500942
211	BAMBI	1.613653	2.196144	1.480652	1.788565	1
212	OSR1	1.784703	2.192671	1.527494	1.794956	1
213	ROBO2	1.771779	1	1.563591	1.864279	2.547887
214	PSG5	1.711991	2.639549	1.597756	1.553418	1
215	RARRES2	1.632339	1	1.609718	2.083456	2.183738
216	PCDHB2	1.497832	1	1.513088	2.180408	2.006454
217	RGS2	1.426901	1.055548	2.177542	1.001126	1
218	LOC730743	1.64674	2.169954	1.437772	1.309118	1
219	EPAS1	1.855435	2.152579	2.169942	1.771992	1
220	RASD1	1.542052	1	1.688727	1.713865	2.167063
222	C3ORF72	1.631804	2.110997	2.166651	1.365314	1
223	EDNRB	2.034003	1	1.712732	1.414047	2.16588
224	DCN	1.736021	1	1.740472	1.489656	2.165106
225	KIAA0367	1.333577	1	1.324392	1.733054	2.164429
226	PTGFRN	1.305388	1	1.461831	1.606624	2.163262
227	SCRG1	1.137386	1	1.10908	1.983506	2.163138
228	PTGS2	1	1.152732	1.001075	1.419964	2.160278
229	EFHD1	1	1.308978	1.055316	2.158003	1.899899
230	IL11	1.564337	1	1.086289	1.19031	2.152981
231	SLC40A1	1.613314	1	1.657035	2.15265	1.966348
232	LOC647444	1.15291	1.328218	1	1.387433	2.147122
233	CAMK2N1	1.873635	1	1.604113	1.9358	2.146981
234	LOC100132564	1.668734	1.346014	1	1.240358	2.145476
235	LOC728946	1.704999	2.144217	1.437381	1.36127	1
236	F2RL2	1.301023	1	1.210799	1.460485	2.141616
237	FZD4	1.653954	1	1.539157	1.949085	2.140827
238	LOC100133277	1	1.559679	1.092554	1.531722	2.140052
239	PSG6	1.592388	2.751437	1.600596	1.540275	1
240	ABCA1	1.585937	1	1.861833	2.139539	1.834684
242	SOX9	1.384621	1	1.407049	1.366282	2.136743
243	HSPB6	1.293679	1	1.12009	1.991674	2.133974
244	FBN2	1.902287	1	1.40683	1.75232	2.132418
245	C16ORF45	1.671979	1	1.602614	1.826197	2.130291
246	CRYAB	1.601219	1	1.369802	2.054874	2.128472
247	C6ORF115	1.773409	2.126124	1.815815	1.215632	1
248	NLGN4Y	1.592934	2.12404	1.998254	1.234387	1
249	CDKN2B	1.193485	1	2.122291	1.665765	1.441976
250	PDGFRL	1.61896	1	1.456672	2.120968	1.938863
251	KCTD12	1.21085	1.677043	1	2.120638	2.01102
252	BIRC3	1.478139	2.120101	1.654269	1.106999	1
253	BMP6	1.674385	1	1.361963	1.79908	2.116601
254	CHURC1	1.421364	1	1.270422	2.047217	2.11584
255	APBB1IP	1.518693	2.113735	1.743086	1.180538	1
256	LOC645638	1.691164	1	1.376839	2.11082	1.45525
257	ABI3BP	1.054244	1.041678	1	2.104917	1.050086
258	PAPPA	1.220251	1	1.5301	2.101986	1.720134
259	LPPR4	1	1.534099	1.013829	2.097118	1.233936
260	ASS1	1.337747	1	1.120129	1.994381	2.096139
262	PRSS35	1.654066	1	2.092605	1.808618	1.288934
263	PPAP2B	1.513813	1	1.384329	1.566102	2.299718
264	RECK	1.470841	1	1.310021	1.911181	2.125405
265	EFEMP1	1.081013	1	1.168962	2.091535	1.381746
266	HMOX1	1.566344	1	1.74554	1.516779	2.08751
267	ERAP2	1.680666	2.086672	1.981379	1.552017	1
268	SOX8	1.232445	1	1.390134	1.419164	2.084882
269	SRGN	1.516822	1.053303	1	2.137566	1.343807
270	STC1	1.473533	1.525718	2.07957	1	1.380079
271	TSPAN13	1.29808	1	1.225306	1.874974	2.101407
272	NGEF	1.52943	1	1.31548	1.649901	2.073251
273	EYA2	1.574234	2.070325	1.806774	1.216112	1
274	PPP1R14A	1.040039	1.614095	1	2.070029	1.020635
275	PDE5A	1.777177	2.06866	2.011819	1.921151	1
276	SERPING1	1.312685	1	1.217725	1.31837	2.065789
277	LOC100132394	1.928356	1.330477	1	1.166734	2.064594
278	RGS4	1.615487	1	1.41677	2.057928	2.06449
279	TSPAN8	1.146808	1	1.286954	1.553001	2.061584
280	C14ORF149	1.302237	1	1.107127	1.756724	2.057625
282	ZNF503	1.559667	1	1.48611	1.597059	2.055934
283	UCHL1	1.352307	1	1.102793	2.055486	1.762723
284	BARX1	1.574788	1	1.264339	1.663742	2.049938
285	FZD6	1.276913	2.047268	1.431044	1.374699	1
286	SLC16A6	1.642858	1	2.046664	1.746392	1.741465
287	SLC16A3	1.479531	2.046663	1.726592	1.30048	1
288	IFITM1	1.299586	1.511211	2.043283	1.142882	1
289	CALB2	1.490767	1.650753	2.037555	1.037354	1
290	COL4A2	1.388761	1.977943	1.18817	2.033886	1
291	P704P	1.416654	1.618327	1	1.498974	2.032729
292	QPCT	1.342987	2.03141	1.652946	1.273012	1
293	KIF20A	1.588363	2.030372	1.33416	1.190238	1
294	LOC390183	1	1.426094	1.081544	1.531061	2.027573
295	LOC648740	1.104449	1.606482	1	1.511866	2.021509
296	LOC728843	1	1.727793	1.251642	1.475895	2.020187
297	NBL1	1.82856	1.97721	2.147261	1.214342	1
298	IGFBP2	1.374727	1	1.339395	2.013086	1.91265
299	FBLN2	1.370924	1	1.438955	1.857047	2.302537
300	PRPS1	1.36303	1	1.069156	1.745295	2.011622
302	CDH10	1.519338	2.009154	1.750165	1.386917	1
303	SLC4A4	1.521623	2.009033	1.469459	1.23475	1
304	PTX3	1.323372	1.236746	1	1.506142	2.008536
305	VCAM1	1.471091	2.007974	1.754668	1.644002	1
306	COL11A1	1.581508	1.609668	1.721789	2.001654	1
307	SCARA5	1.17476	1	1.421212	1.412808	2.000664

## Data Availability

The data that support the findings of this study are available upon request from the corresponding author. The data are not publicly available due to privacy or ethical restrictions.

## Endnotes

^1^Absolutely RNA Nanoprep Kit, Agilent Technologies, CA, USA.

^2^Agilent Technologies, CA, USA.

^3^NanoDrop, Wilmington, DE, USA.

^4^Illumina Inc., CA, USA.

^5^Amersham Biosciences, NJ, USA.

^6^InVitrogen, Camarillo, CA, USA.

^7^Thermo Fisher Scientific, RevertAid First Strand cDNA Synthesis Kit, Waltham, MA, USA.

^8^Roche Diagnostics GmbH, Mannheim, Germany.

^9^Stratagene, La Jolla, CA, USA.

^10^Life Sciences/GIBCO, Paisley, UK.

^11^BD Biosciences, Bedford, MA, USA.

^12^Roche Diagnostics GmbH, Germany.

^13^Versamax, USA.

^14^BD Biosciences, Franklin Lakes, NJ, USA.

## Supporting Information

Supporting Information Figure S1: Gene Ontology (GO) and KEGG pathway enrichment results based on the list of differentially expressed genes (including PENK) in dental-derived MSCs. Enriched terms related to extracellular matrix organization, immune response, and paracrine signaling are highlighted. Adjusted *p*-values are shown for each pathway or term.
