# Supplementary figures and images for: Transcriptomic Profiling of Dental Tissue-Derived Mesenchymal Stem Cells
Source: Stem Cells Int. 2025 Oct 2;2025:4789882. doi: 10.1155/sci/4789882 (PMC12510763; doi:10.1155/sci/4789882)

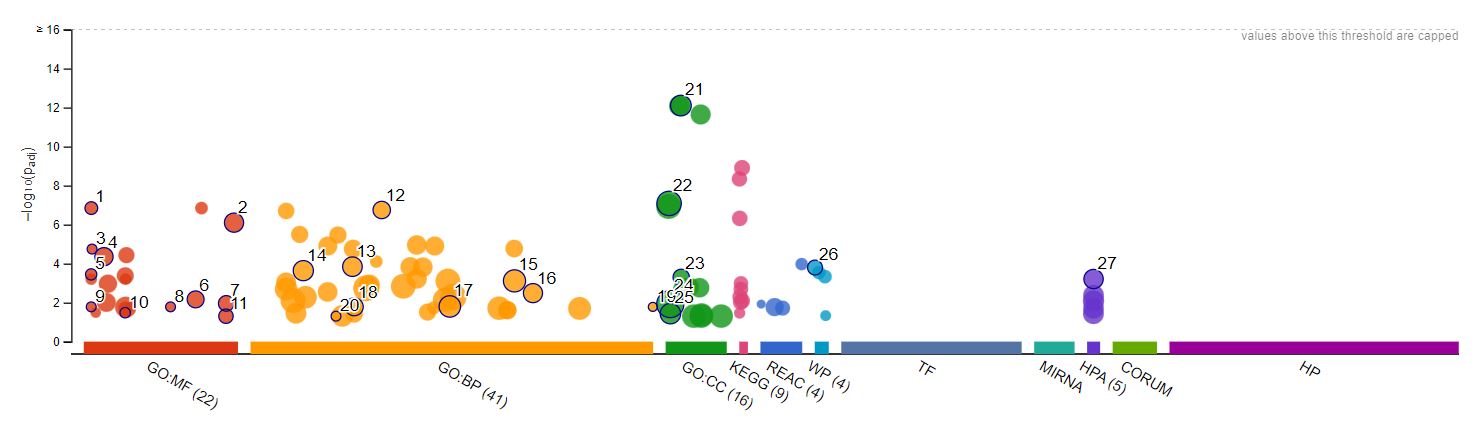

Supplement: Supporting Information — Figure S1: Gene Ontology (GO) and KEGG pathway enrichment results based on the list of differentially expressed genes (including PENK) in dental-derived MSCs. Enriched terms related to extracellular matrix organization, immune response, and paracrine signaling are highlighted. Adjusted p-values are shown for each pathway or term. [file 4789882.f1.zip › Supplementary Figure 1A gene enrich GO.JPG]

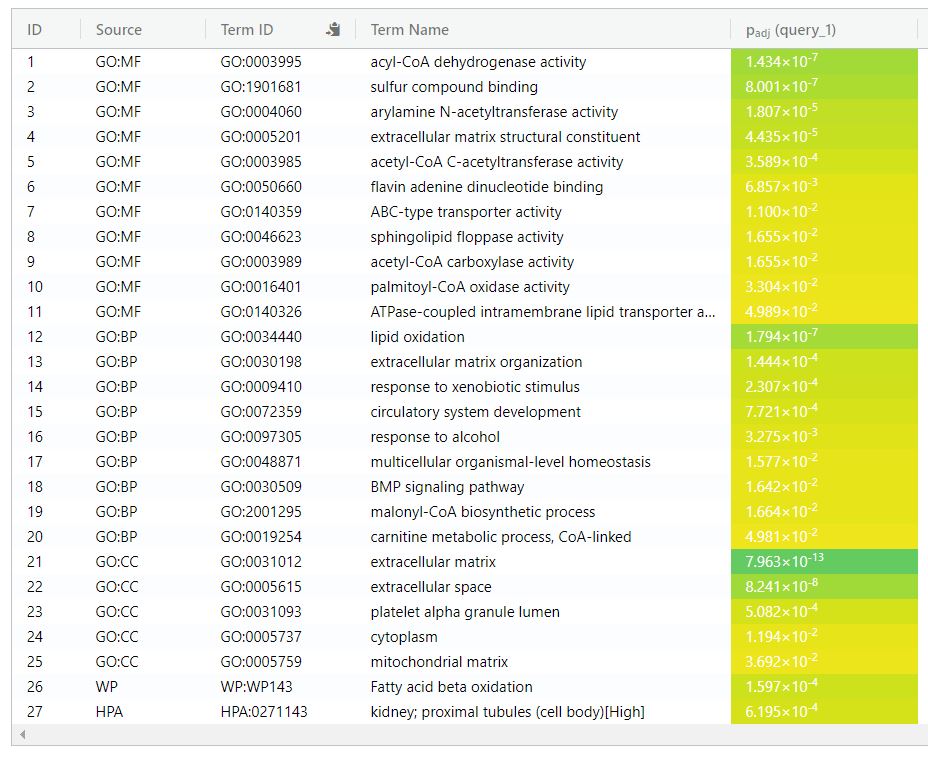

Supplement: Supporting Information — Figure S1: Gene Ontology (GO) and KEGG pathway enrichment results based on the list of differentially expressed genes (including PENK) in dental-derived MSCs. Enriched terms related to extracellular matrix organization, immune response, and paracrine signaling are highlighted. Adjusted p-values are shown for each pathway or term. [file 4789882.f1.zip › Supplementary Figure 1B gene enrich.JPG]
